# Reorganization of the Medical Profession

**Published:** 1905-03

**Authors:** 


### EDITORIAL NOTES AND COMMENTS.

The Business office of The Journal-Record is No. 1007-1008 Century Building.

The Editorial office is 318-319-320 The Prudential.

Address all Business communications to Dr. M. B. Hutchins, Mgr.

Make remittances payable to The Atlanta Journal-Record of Medicine.

On matters pertaining to the Editorial and Original Communications address Dr. Bernard Wolff, Atlanta.

Reprints of original articles will be furnished at cost price. Requests for the same should always be made *on the manuscript.*

We will present, post-paid, on request, to each contributor of an original article, twenty (20) marked copies of The Journal-Record containing such article.

### REORGANIZATION OF THE MEDICAL PROFESSION.

Some months ago we had occasion in commenting upon Virginia's rejection of the proposed plan of reorganization of the medical profession, to outline briefly the scheme of the American Medical Association to effect an organic union of the medical bodies of the United States. The plan was based upon the county medical society as the unit, narrowing into the State medical society and that in turn to the federal organization, the latter to be the supreme body dominating all the lesser organizations. The benefits that were to accrue from such an extensive pooling of interests will be many, the disadvantages those that proceed from a loss of individual liberty and autonomy. The medical profession is called the silent profession and it is silent because it had no spokesman appointed to represent it. Its lack of cohesiveness has rendered representation impractical and it has been submerged. Save in the matter of the public health which it jealously guards, it has no voice in affairs of State or municipality. The result has been that it is when put at issue, scorned and contemned. Quacks flourish in open violation to hardly won laws and the insults of the proprietary medicine advertisements stare it in the face at every hand. There is no one that will not agree that the time is ripe for doctors to band together for common weal and protection and to sound a note that will be heard above the shrill cavilings of the petty politician. But the vital question is can this be done without surrender of privileges that are more valued than those to be bestowed by a changed condition. Should they rather bear the ills they have than fly to others that they know not of? In the contemplated plan of organization the dazzling prospect of drastic purging of the State of all medical offenders is held before our eyes. There will be a sort of medical millennium, when the eclectic and the homeopath and the regular shall lie down together and harmony, like a dove, shall brood over the erstwhile arena of strife. All this glory of coming into the kingdom shall be ours if we assume the yoke. The temptation is singularly acute, but get thee behind me Satan. Self-government, the right of franchise, of free and untrammeled expression are dearer than glittering generalities of this alluring prospect.

The vain gods sigh for the cross and pain,
And the reed that grew, grows never again,
As a reed with the reeds in the river.

We ask again is it necessary to be in the enjoyment of all these benefits that the doctors of Georgia shall relinquish the control of their own affairs and become fused into the general mass. Is there not already at hand and ready organized or partially so, sufficient material which, if properly worked up, is capable of securing all the good that may come from the proposed reorganization plan? Has not Alabama for years, long before this scheme was exploited, furnished an example of self-organization and government that neither sought nor required outside aid or interference?

What Jerome Cochran did for Alabama some Moses may do for Georgia.

It is a wise rule before a reform is adopted to study the precedent. The experiment has not yet been tested, nor a precedent established. If there be defects that work injury, let the defects be discovered at the cost of the rash, while the prudent and cautious bide their time until experience shall have eliminated the mischievous and undesirable elements. Then with all haunting suspicion of sinister motive or ulterior purpose removed, they may heartily join in.

